# Red- and Blue-Light Sensing in the Plant Pathogen Alternaria alternata Depends on Phytochrome and the White-Collar Protein LreA

**DOI:** 10.1128/mBio.00371-19

**Published:** 2019-04-09

**Authors:** Olumuyiwa Igbalajobi, Zhenzhong Yu, Reinhard Fischer

**Affiliations:** a Institute for Applied Biosciences, Department of Microbiology, Karlsruhe Institute of Technology (KIT)-South Campus, Karlsruhe, Germany; Universidad de Córdoba

**Keywords:** blue light, filamentous fungi, light regulation, mycotoxin, phytochrome, plant pathogen, white-collar proteins

## Abstract

The filamentous fungus Alternaria alternata is a common postharvest contaminant of food and feed, and some strains are plant pathogens. Many processes in A. alternata are triggered by light. Interestingly, blue light inhibits sporulation, and red light reverses the effect, suggesting interactions between light-sensing systems. The genome encodes a phytochrome (FphA), a white collar 1 (WC-1) orthologue (LreA), an opsin (NopA), and a cryptochrome (CryA) as putative photoreceptors. Here, we investigated the role of FphA and LreA and the interplay with the high-osmolarity glycerol (HOG) mitogen-activated protein (MAP) kinase pathway. We created loss-of function mutations for *fphA*, *lreA*, and *hogA* using CRISPR-Cas9 technology. Sporulation was reduced in all three mutant strains already in the dark, suggesting functions of the photoreceptors FphA and LreA independent of light perception. Germination of conidia was delayed in red, blue, green, and far-red light. We found that light induction of *ccgA* (clock-controlled gene in Neurospora crassa and light-induced gene in Aspergillus nidulans) and the catalase gene *catA* depended on FphA, LreA, and HogA. Light induction of *ferA* (a putative ferrochelatase gene) and *bliC* (*bli-3,* light regulated, unknown function) required LreA and HogA but not FphA. Blue- and green-light stimulation of alternariol formation depended on LreA. A lack of FphA or LreA led to enhanced resistance toward oxidative stress due to the upregulation of catalases and superoxide dismutases. Light activation of FphA resulted in increased phosphorylation and nuclear accumulation of HogA. Our results show that germination, sporulation, and secondary metabolism are light regulated in A. alternata with distinct and overlapping roles of blue- and red-light photosensors.

## Abstract (precis)

Light controls many processes in filamentous fungi. The study of light regulation in a number of model organisms revealed an unexpected complexity. Although the molecular components for light sensing appear to be widely conserved in fungal genomes, the regulatory circuits and the sensitivity of certain species toward specific wavelengths seem different. In N. crassa, most light responses are triggered by blue light, whereas in A. nidulans, red light plays a dominant role. In Alternaria alternata, both blue and red light appear to be important. In A. alternata, photoreceptors control morphogenetic pathways, the homeostasis of reactive oxygen species, and the production of secondary metabolites. On the other hand, high-osmolarity sensing required FphA and LreA, indicating a sophisticated cross talk between light and stress signaling.

## INTRODUCTION

The genus Alternaria comprises saprophytic and plant-pathogenic filamentous ascomycetes which are cosmopolitan and ubiquitous in nature. They are commonly isolated from dead plant materials, sewage effluents, grains, and indoor air. Alternaria alternata produces multicellular asexual spores (conidia), which are highly melanized. It grows well on food and feed and produces different types of health-endangering mycotoxins, such as alternariol and alternariol monomethyl ether or the perylene derivatives altertoxins (ATX) I, II (also called stemphyltoxin II), III, and stemphyltoxin (1). A. alternata is therefore an economically important mold (2, 3). It is able to cause allergic reactions (asthma) and can be the causative agent of several types of cutaneous and subcutaneous infections (4, 5). A. alternata responds to light, which triggers morphogenetic and metabolic pathways (6). However, the molecular biology of light perception and light signaling in A. alternata is not yet well understood.

Light regulation has been studied extensively in the ascomycetes Neurospora crassa, Aspergillus nidulans, Trichoderma atroviride, Trichoderma reesei, and Botrytis cinerea (7–13). The light response was also analyzed in the zygomycetes Phycomyces blakesleeanus and Mucor cineroides and some basidiomycetes (14–17). In N. crassa, many processes are controlled by blue light, among which are carotenoid biosynthesis and the entrainment of the circadian clock (18). The analysis of blind mutants revealed the white-collar protein 1 (WC-1), a transcriptional regulator which contains a flavin as a chromophore (19–21). A second transcriptional regulator without a chromophore is WC-2, which forms a dimer with WC-1. As a complex (the white-collar complex [WCC]), they act as a positive regulator of light-induced genes (22). A complicated signaling cascade is not necessary, given that the photoreceptor resides in nuclei and directly controls gene expression. In addition, it interacts with the chromatin-remodeling machinery and enables efficient transcription of light-induced genes (23). A second blue-light photoreceptor is the VIVID protein (24, 25). It is involved in photoadaptation, but, differently from the WC proteins, it is only found in some ascomycetes. In Trichoderma spp., the WC orthologues, Blr-1,2 and a VIVID orthologue, ENVOY, fulfill similar functions as in N. crassa (26, 27). The WC signaling pathway is connected to MAP kinase signaling (28, 29). It was shown that the MAP kinase gene *tmk3* is upregulated by light in *T. reesei* and phosphorylated in *T. atroviride* (28, 30). In addition to the blue-light photoreceptor WC-1, two phytochrome genes were identified in the N. crassa genome (31). Deletion of the genes had neither drastic phenotypes on developmental processes nor caused major global changes in gene expression (31, 32). However, there is evidence that phytochrome modulates the WCC activity and is involved in fine-tuning of the balance between sexual and asexual development (33, 34).

Whereas in N. crassa all photoresponses are blue-light responses, A. nidulans mainly responds to red light (32, 35). The red-light induction of conidiation could be reversed by far-red-light exposure, which resembled the phytochrome response in plants. Later, it was shown that a phytochrome (FphA) indeed triggers many processes and controls the expression of a large proportion of the genome (36, 37). Phytochrome was found in the cytoplasm and hubs in the high-osmolarity glycerol (HOG) MAP kinase signaling cascade to transmit the light signal into nuclei (38). In addition, phytochrome fulfills nuclear functions in chromatin remodeling (39). The WC orthologues also exist in A. nidulans but appear to fulfill different functions from those in N. crassa. The WC-1 orthologue LreA acts as an inhibitor of sporulation and is released from light-induced promoters upon illumination (39). The WC-2 orthologue LreB physically interacts with FphA, suggesting cross talk between the blue- and red-light-sensing systems (40). It appears that most fungi respond to light and the blue- or the red-light photoresponse normally dominates.

In Alternaria solani and *B. cinerea,* two plant pathogens, asexual sporulation is inhibited by blue light and promoted by red light, suggesting the action of blue- and red-light photoreceptors (41–45). More interestingly, the blue-light effect could be reversed by red light. We anticipated a similar situation in A. alternata, because it also responds to blue and red light and contains the full repertoire of blue- and red-light photoreceptors (6). Only the WC-1 orthologue LreA had been studied to some extent, because detailed studies were hindered by limited tools in A. alternata and difficulties in generating clean gene deletions. However, recently, the CRISPR-Cas9 technology was established in A. alternata, which opened many new avenues (46). Using this technology, we were able to investigate in this study the roles of FphA, LreA, and HogA in light sensing and the control of sporulation, secondary metabolite formation, and stress adaptation in A. alternata.

## RESULTS

### Interplay between red- and blue-light sensing in A. alternata.

**FIG 1:**
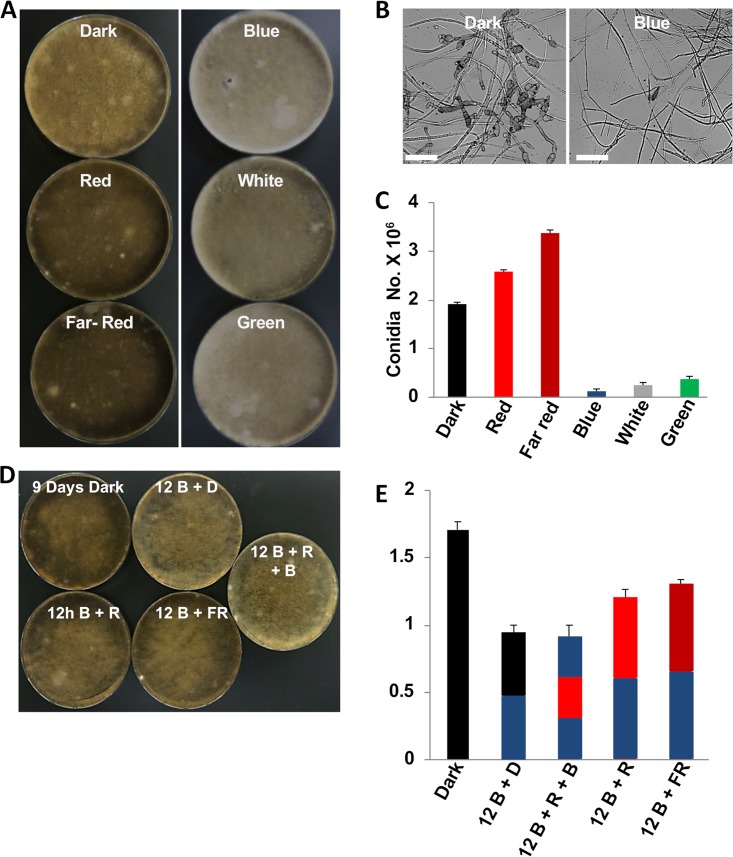
Light sensing and photoreversibility of blue-light inhibition of sporulation. (A) *A. alternata* wild-type conidia (5 × 10^4^) were homogeneously spread onto mCDB plates and incubated at 28°C for 12 days in the dark or at different wavelengths as indicated. (B) Microscopic pictures of spores. Scale bar = 20 µm. (C) Quantification of asexual conidia from the cultures in panel A. (D) Photoreversibility of blue-light inhibition of sporulation by red or far-red light. All cultures were first incubated for 2 days in the dark. The control plate was kept in the dark, whereas the others were exposed for 12 h to blue light followed by dark incubation or 12 h of exposure to red or far-red light and then further in the dark until 12 days. (E) Quantification of the number of conidia from the cultures in panel D. The experiments were repeated three times. The arrow bars represent the standard deviation. D, dark; B, blue; R, red; FR, far red.

Previous studies on the effect of light on sporulation in A. alternata suggested an interesting interplay between blue and red light (6). In order to investigate the effects of light on growth and morphogenesis, A. alternata cultures were incubated at 28°C under different illumination conditions for 12 days (Fig. 1A). Whereas the cultures incubated in the dark and in red and far-red light appeared dark brown, they were pale when illuminated with blue, white, or green light. Melanization of the spores was independent of light, indicating that the change of the color of the cultures on agar plates was due to a reduction in the number of spores (Fig. 1B and C). Green, blue, and white light drastically inhibited sporulation, and more sterile aerial hyphae were produced. Green light was less efficient than blue light (Fig. 1C).

In order to test if red light could reverse the inhibitory effect of blue light, cultures were grown for 2 days in the dark and then exposed for 12 h to blue light, followed by 12 h of incubation in the dark or for the same time under red or far-red light. In one experiment, we treated the culture for 12 h with blue light, followed by 12 h with red light and another 12 h of blue light. After the treatment, all plates were further incubated for 8 days (7.5 days in the last case) in the dark (Fig. 1D). The cultures exposed to red or far-red light produced more spores than did the cultures transferred to the dark after blue-light exposure (Fig. 1E). This suggests that red and far-red light are able to reverse the blue-light photoinhibitory effect. The red-light stimulation could be nullified by another blue-light exposure.

Incubation of center-inoculated A. alternata colonies under 12-h-light/12-h-dark conditions led to rhythmic production of conidia and a ring-like appearance of the colonies (data not shown). Future experiments should reveal if a circadian clock is involved in the rhythmic behavior.

### Phytochrome and the WC-1 orthologue LreA are required for sporulation.

A. alternata possesses photoreceptors for blue (LreA), red (FphA), and green (NopA) light. In addition, other components involved in light regulation in other fungi, such as WC-2 (LreB), velvet, or HogA are conserved. Here, we studied the roles of FphA, LreA, and HogA. The A. alternata phytochrome FphA consists of 1,511 amino acids encoded by a genomic region of 4,536 bp, with two exons and one intron (51 bp). The position of the intron was confirmed by cDNA sequencing. The photosensory domain consists of a PAS domain, a GAF domain, and a PHY domain, and in the C-terminal part, there is a histidine kinase (HK) domain, including an ATPase domain, and a response regulator (RR). The cysteine in the PAS domain and the two nuclear localization signals (NLS) of the phytochrome of A. nidulans are conserved in A. alternata. An N-terminal extension in front of the photosensory domain is also conserved compared to FphA in A. nidulans and N. crassa. The A. alternata *fphA* sequence was used to complement a phytochrome mutation in A. nidulans. Although complementation was not complete, it clearly proofed the functionality and conservation of the gene (Fig. S2). LreA comprises 1,025 amino acids and harbors an LOV domain, two PAS domains, a GATA-type zinc finger domain, and a predicted NLS. It should be noted that the predicted NLS is not required for nuclear localization of WC-1 in N. crassa (47). The open reading frame is disrupted by two introns. The cysteine residue in the LOV domain, required for chromophore binding, is also conserved in A. alternata. The *hogA* gene encodes an open reading frame (ORF) of 1,068 bp interrupted by seven introns and giving rise to a polypeptide of 355 amino acids. It has a protein kinase ATP-binding region as well as a MAP kinase site.

**FIG 2:**
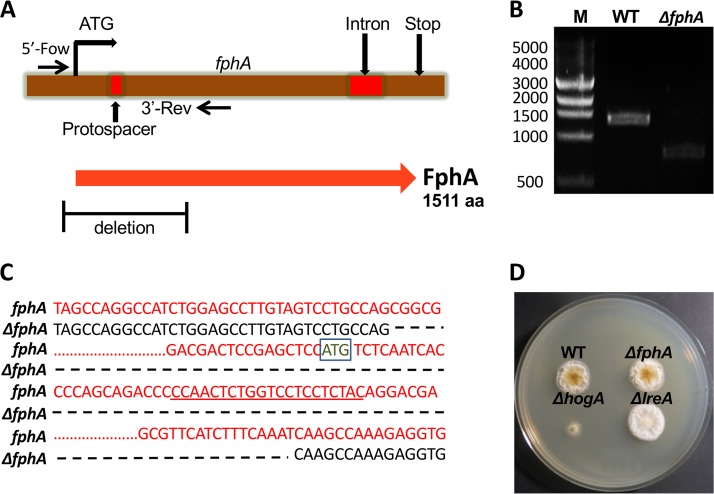
Inactivation of the *fphA* gene using CRISPR-Cas9. (A) Scheme for the inactivation of *fphA*. The primers located upstream and downstream of the protospacer and the protospacer are indicated. The deletion is also shown. aa, amino acids. (B) Confirmation of the CRISPR-Cas9-induced inactivation of *fphA* by PCR using the primers indicated in panel A and genomic DNA as the template. (C) Comparison of the *fphA* mutant sequence (black) with the sequence of the wild type (red) revealed a 528-bp deletion with 467 bp of the *fphA* ORF and 61 bp of the 5′ untranslated region (UTR). The protospacer is underlined. The start codon is boxed. The dashed line shows the missing nucleotides. The dotted line represents 961 nucleotides which were are also missing and not displayed here. (D) Pictures of colonies of the WT and the *fphA*, *lreA,* and *hogA* mutant strains incubated at 28°C for 4 days.

In order to assign a function to FphA, LreA, and HogA in light sensing in A. alternata, we aimed to inactivate the respective genes using the CRISPR-Cas9 technology, which has been established recently in this fungus (46). We chose a protospacer (20 nucleotides) of the respective genes of interest, adjacent to a 3′ AGG protospacer-adjacent motif (PAM) site close to the start of the ORF (Fig. 2A). The protospacer was introduced into plasmid pFC332 by PCR and cloning. The resulting plasmid which contains the Cas9 coding sequence from Streptococcus pyogenes (codon optimized for Aspergillus niger) and the single-guide RNA (sgRNA) targeting the genes of interest (FphA, LreA, and HogA) were used for transformation of the wild-type strain (ATCC 66981). The hygromycin resistance cassette residing in a self-replicating plasmid (AMA plasmid) was used for selection. In the case of *fphA*, 20 hygromycin-resistant transformants were obtained, with two transformants exhibiting a changed phenotype, and for *lreA* out of 30 transformants analyzed, three were positive for the loss of LreA function. In the case of *hogA,* two out of 10 transformants displayed a phenotypic change. Transformants with changed phenotypes were screened for *fphA, lreA,* and *hogA* mutations via PCR (Fig. 2B) and sequencing of the PCR products (Fig. 2C; see also Fig. S1 in the supplemental material). We detected deletions of 528 bp (*fphA*), 3,398 bp (*lreA*), and 535 bp (*hogA*). In all three cases, we assume that the deletions led to complete loss-of-function mutations. The radial growth of the colonies of the *fphA* and *lreA* mutant strains was like that in the wild type (WT), whereas the *hogA* deletion strain grew very badly (Fig. 2D).

10.1128/mBio.00371-19.1 FIG S1 Inactivation of *lreA* and *hogA* using CRISPR-Cas9 technology. (A) Scheme of the *lreA* locus with the protospacer used for inactivation indicated. (B) The primers shown were used to amplify the region in WT and the *ΔlreA* mutant. (C) Scheme of the *hogA* locus with the protospacer used for inactivation indicated. (D) The primers shown were used to amplify the region in WT and the *ΔhogA* mutant. (E and F) Sequence comparison of the WT loci and the corresponding mutants. The dashed line shows the missing nucleotides (4,410 bp for *lreA* and 1,108 bp for *hogA*). The dotted line represents 949 nucleotides, which were not displayed here. Download FIG S1, TIF file, 1.8 MB. Copyright © 2019 Igbalajobi et al. 2019 Igbalajobi et al. This content is distributed under the terms of the Creative Commons Attribution 4.0 International license.

**FIG 3:**
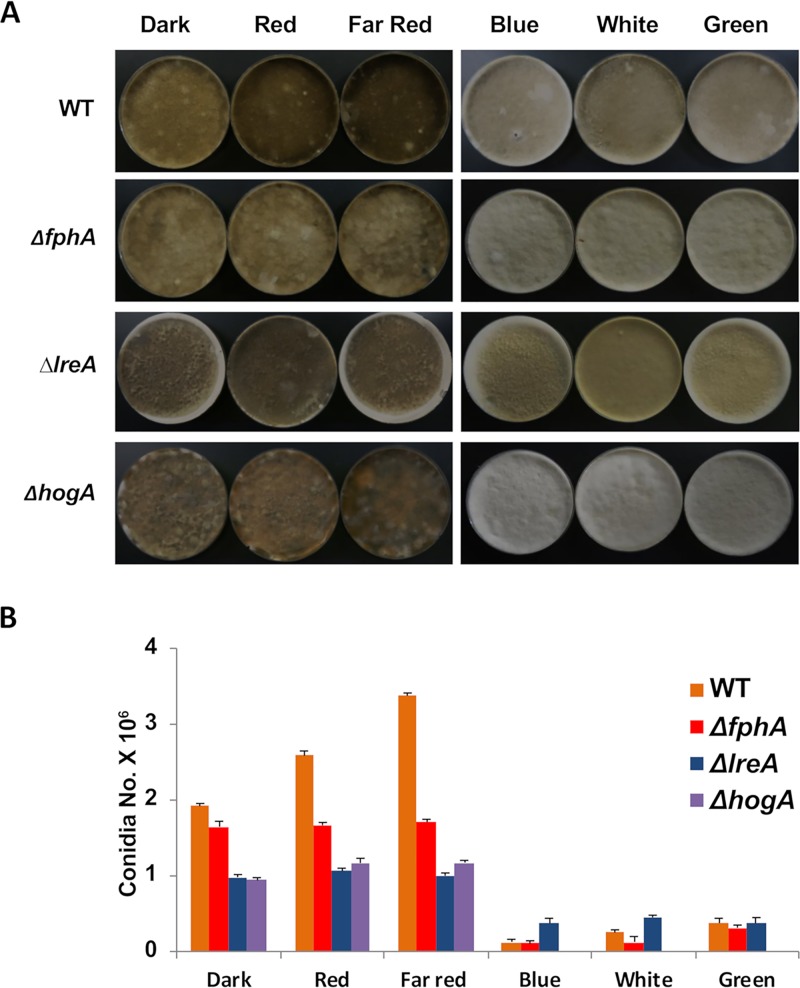
Analysis of the number of conidia in the *A. alternata* wild type (WT) and the *fphA*, *lreA,* and *hogA* mutant strains at different illumination conditions. (A) Colony appearance on mCDB plates inoculated with 5 × 10^4^ conidia evenly spread on the agar surface and incubated at 28°C for 12 days in the dark or illuminated with the light qualities as indicated. (B) Quantification of the conidia produced on the plates in panel A. Three independent plates of each strain were analyzed, and the mean values for the three samples are displayed. The arrow bar represents the standard deviation.

To characterize the role of FphA, LreA, and HogA in sporulation, we investigated the effect of different wavelengths on light regulation of sporulation in the wild-type and corresponding mutant strains (Fig. 3A and B). Sporulation occurred in the dark and was stimulated by red and far-red light. Blue, green, and white light inhibited conidiation. Conidiation was reduced to 86% in the *fphA* mutant strain compared to that in the wild type when incubated in the dark. Light stimulation in red or far-red light was lost in the mutant. The phenotype was rescued after recomplementation with a wild-type copy of *fphA* (Fig. S2). These results, especially the fact that sporulation was reduced in the dark, suggest a positive, but not essential, function of FphA. It also shows that FphA has some function in the dark.

10.1128/mBio.00371-19.2 FIG S2 Recomplementation of the Δ*fphA* and the Δ*lreA* mutant of *A. alternata* (A and B) and recomplementation of the *A. nidulans* Δ*fphA* mutant with *A. alternata fphA* (C). (A and B) Analysis of conidial formation. (C) Expression analysis of the two light-regulated genes *ccgA* and *ccgB* in *A. nidulans*. The values are the mean of three biological repetitions. Statistical analysis was performed with Student’s *t* test, **, *P ≤ *0.01. Download FIG S2, TIF file, 2.1 MB. Copyright © 2019 Igbalajobi et al. 2019 Igbalajobi et al. This content is distributed under the terms of the Creative Commons Attribution 4.0 International license.

In comparison, we expected that blue-light inhibition of sporulation would be released in an *lreA* deletion strain. However, this was not the case. Already in the dark, the *lreA* mutant only produced 51% of the number of spores of the wild type, showing that LreA is important for sporulation independent of light. The reduction in sporulation in the *lreA* mutant strain was restored by recomplementation (Fig. S2). In blue light, sporulation was still inhibited. The *hogA* deletion strain was also tested for sporulation. Spore numbers were reduced to 48%. The plates looked black, suggesting that HogA negatively regulates melanin production in hyphae (48, 49) (Fig. S3). Because we were unable to isolate viable protoplasts from the *hogA* mutant strain, recomplementation of this strain was impossible. Taken together, the results suggest that FphA and LreA act as activators of asexual reproduction in A. alternata, while HogA plays an important role for the general fitness of the organism.

10.1128/mBio.00371-19.3 FIG S3 Analysis of the *A. alternata* wild type (WT) and the *fphA*, *lreA,* and *hogA* mutant strain melanin composition. (A) Pictures of mycelium of the WT and the *fphA*, *lreA,* and *hogA* mutant strains incubated at 28°C (shaking culture) for 7 days. (B) Quantitative analysis of melanin. Melanin was purified with 2% NaOH, and the absorbance at 459 nm was measured using a spectrophotometer. All experiments were done in triplicate. The arrow bar represents the standard deviation. Statistical analysis was performed with Student’s *t* test, *, *P ≤ *0.05; **, *P ≤ *0.01. Download FIG S3, TIF file, 1.1 MB. Copyright © 2019 Igbalajobi et al. 2019 Igbalajobi et al. This content is distributed under the terms of the Creative Commons Attribution 4.0 International license.

### Light inhibition of germination and the role of phytochrome.

**FIG 4:**
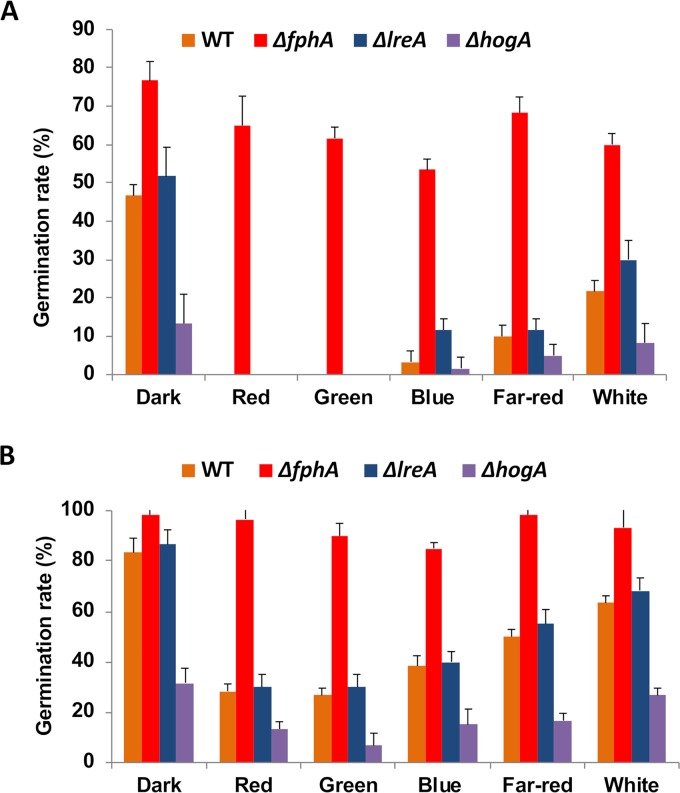
Analysis of the germination rates of conidia from wild-type and different mutant strains in the dark and under different light conditions. (A and B) A conidial suspension (10^5^ per ml) was incubated in minimal medium on a coverslip and the number of germinated conidia (*n* = 100) determined microscopically after 2 h (A) and 3 h (B).

In order to test if germination of spores was affected by light, we tested the effect of different light qualities on the germination rate. In complete medium at 28°C, germination occurred instantly, and no difference under different light conditions or in different mutants (besides the *hogA* mutant) was observed. However, in minimal medium with 1% glycerol (instead of glucose) and incubation at 22°C, we found that after 2 h, ca. 50% of the spores had germinated, and after 3 h, nearly 100% had produced a germ tube. In comparison, when spores were incubated under red light, only 30% germinated after 3 h. Also, far-red, blue, and green light inhibited germination (Fig. 4). This behavior is similar to other fungi (50, 51). However, in A. nidulans, far-red light was more effective than red light.

Deletion of the phytochrome gene released the repression under all conditions. Germination was enhanced already in the dark, showing that FphA plays a role not only in light perception. The deletion of *lreA* did not release the blue-light inhibition completely, suggesting the presence of additional blue-light-sensing systems (Fig. 4).

### Blue- and green-light induction of alternariol biosynthesis depends on LreA.

**FIG 5:**
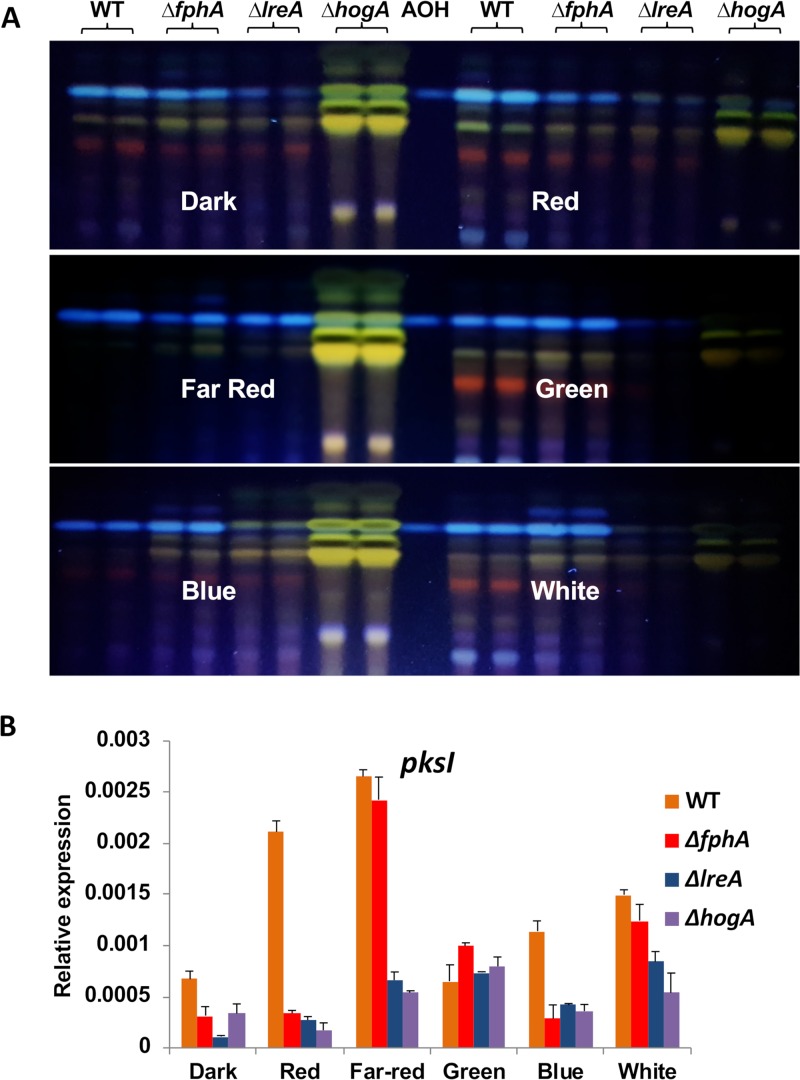
Secondary metabolite analysis by thin-layer chromatography. (A) Extracts from the WT and mutant strains grown for 7 days on mCDB agar plates at 28°C in darkness or under red-, far-red-, green-, blue-, or white-light illumination, respectively. A total of 5 × 10^4^ conidia were spread evenly on the agar surface. An AOH standard was applied on the TLC for comparison. (B) Expression analysis of the *pksI* gene involved in AOH production. Liquid mCDB medium was inoculated with conidia and grown without shaking for 7 days. Mycelia were harvested and processed for RNA extraction and real-time PCR analysis. The mean of the results from three biological replicates and three technical replicates is shown. Expression was normalized to the expression of the *H2B* gene.

Because A. alternata is a producer of a wide variety of different secondary metabolites with different toxicological properties, among which are the mutagenic mycotoxins alternariol (AOH) and altertoxin (ATX) (52), we analyzed the effect of the mutation of *fphA, lreA,* or *hogA* on the amount of secondary metabolites produced under different light conditions. We inoculated modified Czapek Dox broth (mCDB) agar plates with conidia of the WT and the mutant strains and incubated them for 7 days. Both mycelia and agar medium were extracted with ethyl acetate and analyzed by thin-layer chromatography (TLC). Purified AOH was used as a standard. In the WT, the secondary metabolite profile changed with different light sources. In red light, the AOH amount appeared to be increased, whereas most other bands appeared similar to the dark control. Under far-red and blue light, the amount of most bands was reduced, with the exception of AOH. Green light caused an increase in most bands, with the exception of AOH. White light had no drastic effect. In the dark, the secondary metabolite profiles of the WT and the *fphA* and *lreA* mutant strains looked very similar, with drastically reduced amounts of AOH in the *lreA* mutant strain. In the *hogA* mutant strain, the profile was different, with a large increase in the amount of a yellow band. The *fphA* and *lreA* mutant strains exhibited reduced production of AOH under far-red, red, and dark conditions, respectively (Fig. 5A and B). Our results suggest an activating function of LreA under all light conditions, with the exception of far-red light. It was also interesting to notice that the increase in the amount of the yellow band in the *hogA* mutant was reduced when illuminated with green light. In order to further analyze the effects, we studied the expression of the polyketide synthase (PKS) gene responsible for alternariol formation, *pksI* (53). The expression was induced by red and far-red light and to a lower extent by blue light. The red-light response was lost in the Δ*fphA, ΔlreA,* and Δ*hogA* mutants. In far-red light, induction was still observed in the Δ*fphA* mutant. This was unexpected and may suggest some additional far-red light effect (Fig. 5B).

### Phytochrome, LreA (WC-1), and HogA regulate gene expression.

**FIG 6:**
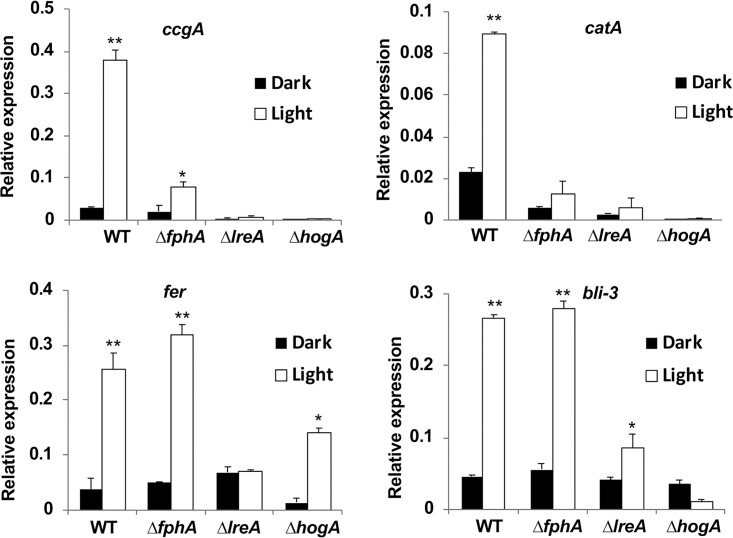
Expression analysis of selected light-regulated genes in the WT and mutant strains. Mycelia were grown in mCDB medium for 36 h at 28°C and then either kept in the dark or exposed for 30 min to white light. RNA was isolated and transcript levels determined by real-time PCR using specific primers for the genes analyzed. The expressions were normalized using *H2B*. All experiments were done in triplicate (three biological and three technical replicates). The arrow bar represents the standard deviation. Statistical analysis was performed with Student’s *t* test, *, *P ≤ *0.05; **, *P ≤ *0.01.

The red- and blue-light receptors in A. alternata appear to play similar but also unique roles compared to those in N. crassa or A. nidulans. Therefore, the next question was about their roles at the gene level. To this end, we investigated light induction of four genes. The first candidate was *ccgA*. Light induction of *ccgA* or *ccg-1* depends on phytochrome and *hogA* in A. nidulans and on HogA and WC-1 and WC-2 in N. crassa (38, 54–56). Mycelia of the WT and the *fphA, lreA,* and *hogA* mutant strains were grown in the dark at 28°C for 36 h and exposed to white light for 30 min. After RNA extraction, the amount of mRNA transcript was determined. Indeed, *ccgA* was induced to about 13-fold in the light compared to the dark (Fig. 6). In the *fphA* mutant strain, light induction was reduced to 20% and to even less in the *lreA* and *hogA* mutants. Hence, FphA, HogA, and LreA all appear to be positive regulators for *ccgA* light induction. In A. nidulans, only FphA is required for *ccgA* induction, and in N. crassa, single mutations in either *wc-1* or *wc-2* resulted in a loss of expression of *ccg-1* (54). The next candidate was the catalase gene *catA*, whose expression is also described in the Discussion. The regulatory behavior of *catA* appeared to be very similar to the regulation of *ccgA*. The same was true for a gene whose translational product displays similarity to short-chain dehydrogenases/reductases (*AAT_PT02522*) (Fig. S4). The gene was identified in A. nidulans in RNA sequencing (RNA-seq) approaches to isolate light-regulated genes (our unpublished data). Next, we tested the light regulation of *ferA*. This gene displays similarity to the *fer* gene of N. crassa, where it encodes a ferrochelatase, an enzyme that catalyzes the formation of heme. In N. crassa, the gene is strictly regulated by blue light and by WC-1 (57). In A. alternata, it was strongly induced by white light. The induction was independent of FphA but strictly dependent on LreA. In the *hogA* mutant strain, light induction was still observed, although the amount of transcript was reduced compared to that in the WT. At last, we tested an orthologue of N. crassa *bli-3* (blue light induced-3; unknown function) (58). Light induction of this gene was also independent of FphA and dependent on LreA. Light induction was lost in the *hogA* mutant strain. The results indicate a complex regulatory network for light-regulated genes in A. alternata, with similarities to N. crassa and A. nidulans.

10.1128/mBio.00371-19.4 FIG S4 Expression analysis of a putative short-chain dehydrogenase in *A. alternata* in the dark and under white-light illumination. The values are the mean of three biological repetitions. Statistical analysis was performed with Student *t* test, **, *P ≤ *0.01. Download FIG S4, TIF file, 0.8 MB. Copyright © 2019 Igbalajobi et al. 2019 Igbalajobi et al. This content is distributed under the terms of the Creative Commons Attribution 4.0 International license.

### Multistress responses in A. alternata are dependent on FphA, LreA, and HogA.

**FIG 7:**
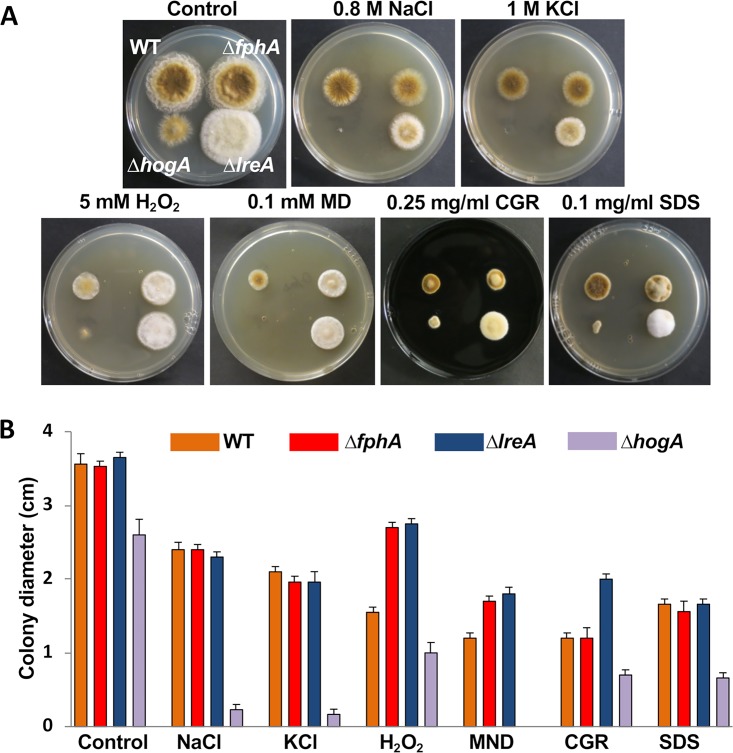
Role of *fphA, lreA* and *hogA* in stress response. (A) Growth of WT and the three mutant strains under different conditions (mCDB plates supplemented with 0.8 M NaCl, 1 M KCl, 5 mM H_2_O_2_, 1 mM menadione (MND), 0.25 mg/ml Congo red (CGR), or 0.1 mg/ml SDS) incubated for 4 days at 28°C in the dark. (B) Quantification of the colony diameter from the colonies in (A). The experiments were repeated three times and the arrow bar represents the standard deviation.

In N. crassa*, T. atroviride, B. cinerea,* A. nidulans, and A. fumigatus, links between light and stress signaling were shown (28, 38, 50, 59, 60). In order to assign a role for FphA, LreA, and HogA in the modulation of stress responses in A. alternata, we investigated the effect of the inactivation of these genes on medium supplemented with osmotic, oxidative, and cell wall-degrading agents incubated at 28°C for 4 days in the dark. The *fphA*- and *lreA*-deletion strains showed no difference in the resistance to osmotic stress with NaCl and KCl or the cell wall stress compounds Congo red and SDS compared to WT. However, both deletion strains displayed enhanced resistance to H_2_O_2_ and menadione. The *hogA* mutant strain was highly sensitive to all substances (Fig. 7A and B). There were no significant differences when the experiment was performed under different light conditions (results not shown).

**FIG 8:**
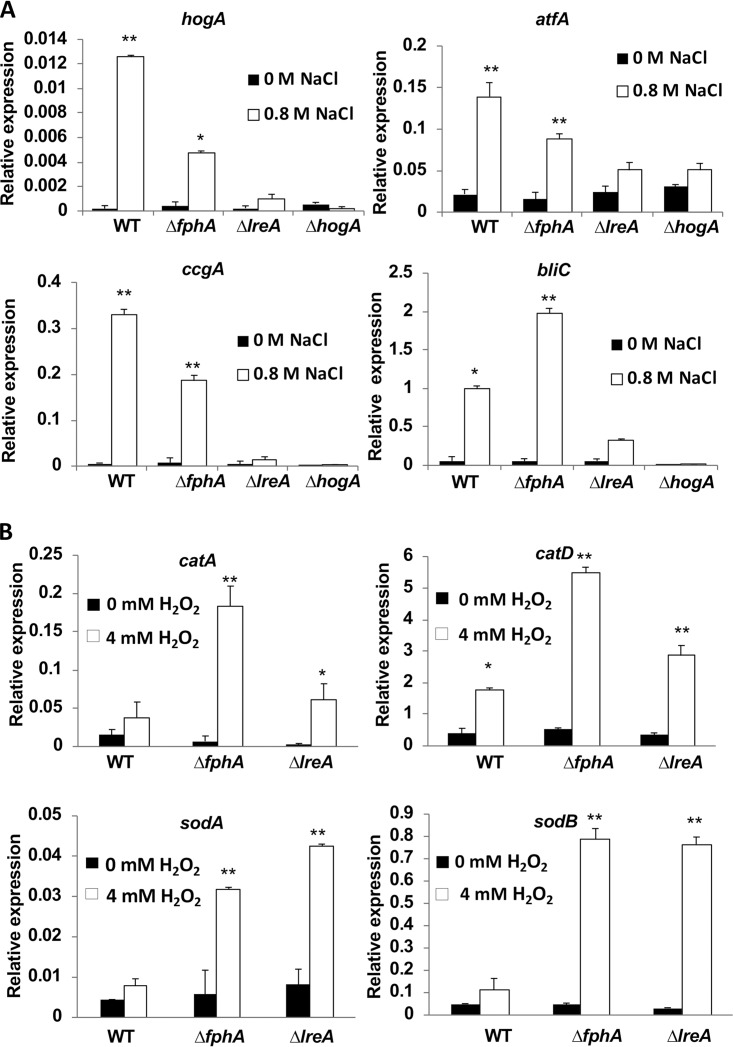
Expression analyses of light and/or osmotic stress regulated genes and the role of FphA, LreA, or HogA. (A) Expression analysis after osmotic shock treatment. RNA was extracted from cultures of the WT and Δ*fphA,* Δ*lreA,* and Δ*hogA* mutant strains grown on mCDB medium for 18 h (shaking) at 28°C and then supplemented with 0.7 M NaCl and further incubated for 30 min. (B) Expression analysis after oxidative stress application. Strains were grown on mCDB medium for 18 h (shaking) at 28°C and then supplemented with 4 mM H_2_O_2_ and further incubated for 30 min. (A and B) mRNA transcript levels were determined by real-time PCR using specific primers for the genes analyzed. The data were normalized using *H2B*. All experiments were done in triplicate. The arrow bar represents the standard deviation. Statistical analysis was performed with Student’s *t* test, *, *P ≤ *0.05; **, *P ≤ *0.01. CGR, Congo red.

Previous reports suggested a cross talk between phytochrome, the white-collar complex, and the HOG MAP kinase cascade (28, 38). Therefore, we analyzed the expression of *hogA, atfA, bliC,* and *ccgA* in the WT and mutant strains in the presence of 0.8 M NaCl compared to that in standard media. The transcript levels of *hogA, atfA,* and *ccgA* were reduced in the *fphA*- and *lreA*-mutant strains compared to those in the WT. The *lreA* mutant strain exhibited reduced expression for *bliC,* which was independent of FphA. The loss of *hogA* resulted in a complete loss of the induction of *ccgA* and *bliC* (Fig. 8A).

In order to better understand the enhanced oxidative stress resistance of the *fphA* and the *lreA* mutant strains, the transcript levels of two catalase (CAT) and four superoxide dismutase (SOD) genes were analyzed in the presence of 4 mM H_2_O_2_. The transcript levels of all tested genes were upregulated more than 6-fold in the *fphA* and *lreA* mutant strains compared to those in the WT (Fig. 8B and S5). Taken together, our results suggest a role for FphA and LreA in the oxidative stress response that is independent of light.

10.1128/mBio.00371-19.5 FIG S5 Expression analysis of the two superoxide dismutase genes *sod-3* and *sod-5* in the presence of 4 mM H_2_O_2_ in WT and the *ΔfphA* and Δ*lreA* mutant strains. The values are the mean of three biological repetitions. Statistical analysis was performed with Student’s *t* test, **, *P ≤ *0.01. Download FIG S5, TIF file, 1.5 MB. Copyright © 2019 Igbalajobi et al. 2019 Igbalajobi et al. This content is distributed under the terms of the Creative Commons Attribution 4.0 International license.

**FIG 9:**
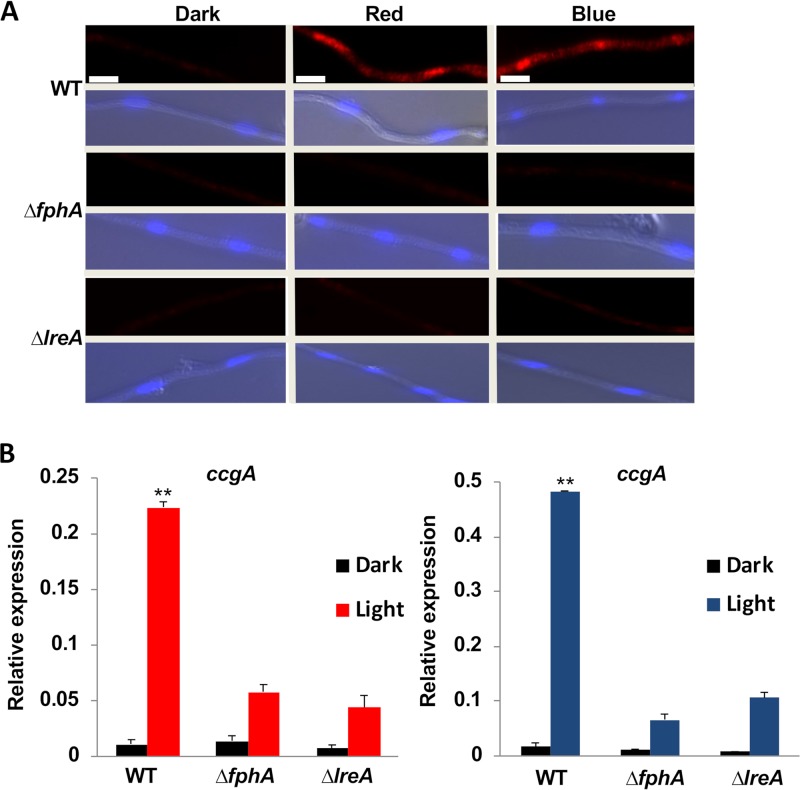
Immunofluorescence analysis of phosphorylated HogA. (A) Microscopic pictures of germlings of WT and mutant strains incubated in the dark or under red or blue light. Conidia were germinated for 3 h at room temperature and processed for immunostaining. Scale bar = 5 µm. (B) Expression analysis of *ccgA* in the dark and under red- or blue-light conditions. Hyphae were grown for 36 h at 28°C and then exposed for 30 min to red or blue light. RNA was extracted and *ccgA* expression quantified by real-time PCR using *H2B* for normalization. The mean of the results from three biological and three technical replicates is shown. Statistical analysis was performed with Student’s *t* test, **, *P ≤ *0.01.

Because of the interconnection between light and stress signaling, we anticipated that the MAP kinase HogA plays a central role in light and stress signaling. In order to test this hypothesis in A. alternata, the phosphorylation level of HogA was studied by immunofluorescence (Fig. 9). Conidia were germinated for 3 h on coverslips in the dark, exposed for 5 min to red or blue light, and processed for immunostaining using the antiphospho-p38 MAP kinase antibody, which has been used to detect A. nidulans phosphorylated SakA (38, 61). A very weak red fluorescent signal was detected in the dark. After illumination with red or blue light, the signal increased very rapidly, and fluorescence was detected in the cytoplasm and enriched in nuclei. After 15 min of illumination, the signal was much weaker again, suggesting a transient activation of *hogA.* The stimulation of the signal was not observed in the *ΔfphA* or Δ*lreA* mutant strains (Fig. 9A). The results are in agreement with expression analyses of *ccgA* after illumination with red or blue light (Fig. 9B).

## DISCUSSION

**FIG 10:**
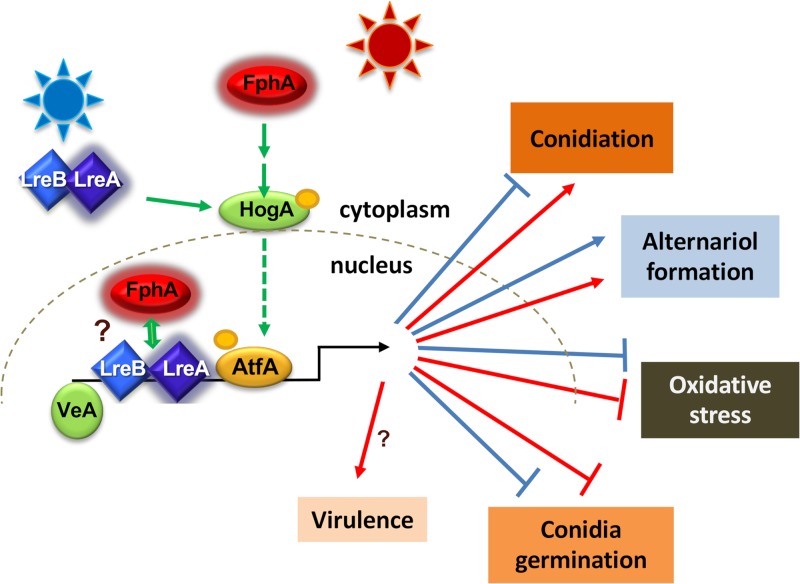
Scheme of light regulation in *A. alternata*. Red light is sensed through phytochrome and involves the HOG MAP kinase pathway for signal transduction into the nucleus. Phosphorylation of HogA is stimulated by red and blue light and depends on phytochrome and the WC complex. The photoreceptors and the WC complex along with VeA play probably also a role in chromatin remodeling, which was not studied in this paper. Binding of the WC complex and of VeA to the promoter of light-regulated genes has been shown in *A. nidulans* but not yet in *A. alternata*. Many morphological and physiological processes are controlled in different ways by blue or red light.

A. alternata is an important food contaminant with an interesting photobiology regulating morphogenetic pathways and physiological aspects. In related fungi, it was reported that there are two distinct phases of photosporogenesis. In the first, or inductive, phase, light stimulates conidiophore formation. However, the second, or terminal, phase, when conidia are produced, occurs only in the dark (62, 63). In comparison to other model organisms such as A. nidulans or N. crassa, light regulation in A. alternata appears to be very complex (Fig. 10). At the molecular level, both photoreceptors, phytochrome and the white-collar orthologue LreA, have activating functions with respect to the induction of sporulation. Whereas LreA is essential, FphA is only required for high levels of spore production. Interestingly, both photoreceptors play roles in the dark. The dark functions resemble the ones described in other systems. In A. nidulans, the deletion of *lreA* led to reduced levels of cleistothecial formation in the dark (40), and in N. crassa, the WC-1 protein is required for clock functioning in the dark (18). Another interesting observation was the fact that far-red light had the same effect as red light. If red light causes photoconversion of FphA into the far-red form (Pfr) and far-red light its reversion back to the red-light form (Pr), one would expect that FphA in the Pfr form is inactive and the strain should respond as in the dark. This however, is not the case. This apparent contradiction has been observed before in A. nidulans*, B. cinerea,* and Beauveria bassiana (9, 51, 64). It has to be considered that long-term illumination may cause effects other than just photoconversion of the chromophore due to different protein interactions and stability of the protein. In plants, the far-red light effect was explained by a combination of Pfr-dependent nuclear import and degradation in the nucleus (65). In fungi, the mechanism for far-red light responses has yet to be investigated.

With respect to individual genes, there are light-induced genes like *ccgA* which are also positively regulated by both photoreceptors, and again, light induction appears to be strictly dependent on LreA and only to some extent on FphA. In addition, the *ferA* and *bliC* genes are also light induced in the absence of FphA, but their induction depends on LreA. This regulatory scheme is different from that in A. nidulans, where LreA binds in the dark to the promoter of *ccgA* and might have a repressing function on *ccgA* light induction. It is also different from N. crassa, where *ccg-1* light induction only depends on WC-1. These observations suggest an interesting regulation and action of the photoreceptors at the molecular level in A. alternata. One possible scenario could be that LreA in combination with the white-collar orthologue LreB acts as a transcriptional activator in addition to FphA as a regulator of the chromatin structure. Chromatin remodeling could be achieved probably by different cues. In our case, it could be that in the case of *ccgA,* FphA-dependent chromatin remodeling is very important for gene induction, whereas in the case of *ferA* or *bliC,* this could be achieved by other modifiers active under the relevant experimental conditions. Or, LreA itself might control the chromatin structure in that case. It has been shown in A. nidulans and N. crassa that FphA and WC-1 interact with chromatin-remodeling enzymes (23, 39).

We also tested the expression of some putative developmental regulators. One developmental gene from A. nidulans, *veA,* was already studied in A. alternata (66). Although the A. nidulans central regulator BrlA was not identified, we found orthologues of many other proteins from A. nidulans and other fungi. Their expression was stimulated after 1 h of illumination with white light, and the stimulation depended in most cases on *fphA* and *lreA* (Fig. S6). After longer exposure (4 h) to white light, no difference in the transcript levels between the dark and the light samples was obvious, suggesting transient gene induction (data not shown). The findings appear to be contradictory to the inhibition of conidial formation by light (Fig. 1). However, it has to be considered that the first phase of conidiation is stimulated by light (see above). More experiments are required to unravel the complex regulation of development and the interplay between the developmental genes.

10.1128/mBio.00371-19.6 FIG S6 Expression analysis of several developmental genes from different *A. nidulans*, *B. cinerea*, and *B. bassiana* strains. Mycelium was grown in mCDB medium for 36 h at 28°C and then either kept in the dark or exposed for 1 h to white light. RNA was isolated and transcript levels determined by real-time PCR using specific primers for the genes analyzed. The expressions were normalized using *H2B*. All experiments were done in triplicate (three biological and three technical replicates). The arrow bar represents the standard deviation. Download FIG S6, TIF file, 2.1 MB. Copyright © 2019 Igbalajobi et al. 2019 Igbalajobi et al. This content is distributed under the terms of the Creative Commons Attribution 4.0 International license.

An important player in red-light regulation and stress signaling in A. nidulans is the HOG MAP kinase SakA (38, 61, 67). There is also evidence for a link between blue-light signaling and the transcriptional and posttranslational regulation of the MAP kinase Tmk3 in Trichoderma atroviride and in N. crassa (28, 30, 68). In A. alternata, high-osmolarity induction of the transcription of *hogA* and the transcription factor gene *atfA* depended on *lreA* and partially on *fphA*. Interestingly, the salt induction of *ccgA* and *bliC* showed a similar regulation. The models developed for A. nidulans cannot explain this regulation. Light and salt signaling should be separated in A. nidulans. It was reported that the MAP kinase SakA was still able to shuttle into nuclei upon salt stress even in the absence of FphA (38).

One interesting observation was the fact that the lack of either photoreceptor, FphA or LreA, led to increased resistance of A. alternata toward oxidative stress. We found that several catalases and superoxide dismutases were upregulated in *fphA* or *lreA* mutant strains. Such a link between light sensing and stress adaptation has been noted before in *T. atroviride, B. cinerea,* N. crassa, B. bassiana, A. nidulans, and A. fumigatus (28, 30, 50, 64, 69). However, in contrast to A. alternata, in *B. cinerea,* deletion of the WC-1 orthologue increased the sensitivity toward oxidative stress. The increased stress resistance in A. alternata was observed in the dark. In nature, the exposure to oxidative stress is linked to light, and light has been considered to be an early alerting system for stressful conditions (7, 69). However, in light (independent of the wavelength), no difference was observed with respect to colony growth in the presence of H_2_O_2_ (data not shown), despite the fact that the catalase gene *catA* was transcriptionally upregulated after light exposure. These results suggest a repressing function of FphA and LreA on the oxidative stress response of A. alternata. On the other hand, FphA and LreA were required for light induction of *catA,* suggesting that light prepares the fungus for oxidative stress, but once the stress is effective, the two proteins appear to modulate the response. This points to a sophisticated feedback regulatory network between light and stress.

## MATERIALS AND METHODS

### Culture conditions and harvesting of conidia.

A. alternata and A. nidulans strains are listed in Table 1. A. alternata ATCC 66981 cultures were grown on modified Czapek Dox broth (mCDB) agar, unless otherwise specified, and incubated for 1 to 12 days at 28°C. A. nidulans was grown as described previously (70). For white-light experiments, a 10-W energy-saving lamp (Flare Energy) was used; for red-, far-red-, blue-, and green-light conditions, light-proof ventilated boxes with wavelength-specific (680, 740, 450, and 550 nm, respectively) light-emitting diodes (LEDs) were used. All plates were inoculated with conidial suspensions. For quantification, conidia were harvested in sterile H_2_O, filtered for separation from the mycelium, and concentrated by centrifugation. The number of conidia was counted in a Neubauer counting chamber.

**TABLE 1:** *A. alternata* and *A. nidulans* strains used in this study

Strain	Genotype or description	Source
*A. alternata*		
ATCC 66981	Wild type	Christopher Lawrence (Blacksburg, VA)
SOI1	*ΔfphA528*	This study
SOI3	*ΔlreA3398*	This study
SOI4	*ΔhogA535*	This study
SOI5	*ΔfphA528* complemented with *Alternaria fphA*	This study
SOI6	*ΔlreA3398* complemented with *Alternaria lreA*	This study
*A. nidulans*		
SJP1	*pyrG89 ΔargB*::*trpCΔB pyroA4 ΔfphA*::*argB veA^+^*	Purschwitz et al. (40)
SZY157	*pyrG89 ΔargB*::*trpCΔB pyroA4 ΔfphA*::*argB veA^+^ Alt. fphA*	This study
SKV103	*pyrG89; pyroA4; veA^+^*	Vienken et al. (73)

### Germination assay.

For the germination experiment, 1 × 10^5^ fresh conidia of the WT and *fphA* and *lreA* mutant strains were inoculated into liquid minimal medium containing 1% glycerol. Four hundred microliters of the suspension was applied to a sterile coverslip, placed in a 10-mm petri dish, and incubated at 22°C for 2 and 3 h in the dark or under light conditions (blue light [450 nm], green light [550 nm], red light [700 nm], far-red light [740 nm], or white light). To determine the rate of germination, a total of at least 100 spores per sample were examined microscopically. All experiments were repeated at least three times.

### Protoplast transformation of A. alternata.

Fungal spores were harvested from an mCDB culture plate and inoculated into 100 ml liquid mCDB medium (4% glucose, 0.1% yeast extract, 0.1% NaNO_3_, 0.025% NH_4_Cl, 0.1% KH_2_PO_4_, 0.025% KCl, 0.025% NaCl, 0.05% MgSO_4_ [MgSO_4_·7H_2_O], 0.001% FeSO_4_, 0001% ZnSO_4_, 1.5% agar) for overnight cultivation at 28°C and 150 rpm. The mycelium was harvested by filtering, washed with 0.7 M NaCl, and digested in a Kitalase (Wako Chemicals) suspension (150 mg in 15 ml of 0.7 M NaCl) for 1 h with gentle shaking at 120 rpm at 30°C. Protoplast quality and quantity were checked microscopically. Protoplasts were separated from cell fragments by filtering through two layers of Miracloth and precipitated at 2,430 rpm for 10 min at room temperature. The Kitalase solution was discarded, and protoplasts were washed once with ice-cold 0.7 M NaCl and resuspended in 100 µl STC (1 M sorbitol, 50 mM CaCl_2_, 50 mM Tris-HCl [pH 8]). Ten micrograms of plasmid DNA was added to the protoplasts, followed by a 10-min incubation on ice. DNA uptake was induced with a heat shock at 42°C for 5 min, and after a 5-min incubation step on ice, 800 µl of 40% polyethylene glycol 4000 (PEG 4000), 50 mM Tris-HCl (pH 8), and 50 mM CaCl_2_ were added to the protoplasts, followed by 15 min of incubation at room temperature. The suspension was mixed with 50 ml warm regeneration medium and split into two petri dishes. After overnight incubation at 28°C, the transformation plates were overlaid with 15 ml warm regeneration medium containing hygromycin (80 µg/ml).

### CRISPR-Cas9 plasmid construction.

The CRISPR-Cas9 vectors with specific sgRNA genes, containing the respective protospacer sequences as well as a 6-bp inverted repeat of the end of the protospacer to complete the hammerhead cleavage site, were generated in a single cloning step. New protospacer sequences were inserted into the linearized pFC332 vector by combining two PCR fragments amplified from plasmid pFC334 and the pFC332 vector in a NEBuilder reaction (New England BioLabs, Frankfurt, Germany). The primers, which contain the variable regions, used to generate the sgRNA gene fragments were obtained from MWG Eurofins and are listed in Table S1. The amplified fragments were flanked by 30-bp complementary sequences to each other and the linearized vector in order to generate the functional vectors in a single NEBuilder reaction. The fragments were amplified from pFC334 with proofreading polymerase Q5 (NEB) by a touchdown PCR program (denaturation, initial step for 3 min at 98°C; all following denaturation steps for 20 s at 98°C; annealing, 5 cycles at 67°C for 20 s, 5 cycles at 65°C for 20 s, 25 cycles at 63°C for 20 s; and elongation, 10 s at 72°C). Standard reaction mixture volumes were 50 μl, including 1 U Q5 reaction buffer, 200 μM dinucleoside triphosphates (dNTPs), 0.5 μM primers, 1 U Q5, and 100 ng of plasmid DNA. Plasmid pFC332 was linearized using PacI and assembled with the PCR fragments, following the NEBuilder protocol. Escherichia coli transformation and plasmid isolation were done according to standard protocols (71).

10.1128/mBio.00371-19.7 TABLE S1 Oligonucleotides used in this study. The red letters indicate the protospacer sequences. Download Table S1, DOCX file, 0.02 MB. Copyright © 2019 Igbalajobi et al. 2019 Igbalajobi et al. This content is distributed under the terms of the Creative Commons Attribution 4.0 International license.

### Assays of cellular stress.

Fresh conidia of different strains were collected from cultures grown on mCDB plates at 28°C for 12 days. Drops of conidial suspensions containing 5,000 conidia of the WT or *fphA*, *lreA,* and *hogA* mutant strains were inoculated mCDB supplemented with NaCl (0.8 M) and KCl (1 M) for osmotic stress. To assay tolerance to oxidative stress, the plates were supplemented with H_2_O_2_ (5 mM) and menadione (1 mM). To analyze the tolerance to cell wall-degrading agents, Congo red (0.25 mg/ml) and SDS (0.1 mg/ml) were added to the medium. All cultures were incubated at 28°C for 4 days. The experiments were carried out in triplicate.

### Melanin assay.

The melanin composition in A. alternata wild type (WT) and the *fphA*, *lreA,* and *hogA* mutant strains was analyzed on mCDB liquid medium after incubation at 28°C (shaking culture) for 7 days. Mycelia of the respective strains were filtered and frozen in liquid nitrogen. The frozen mycelia were ground into powder, suspended in NaOH solution, and boiled at 100°C for 2 h. The solution was acidified to pH 2.0 with 5 M HCl and centrifuged at 10,000 × g for 20 min. The resulting melanin solution was dissolved in 2% NaOH, and the absorbance at 459 nm was measured using a spectrophotometer (72).

### RNA isolation and quantitative real-time PCR.

Conidia were inoculated with a loop on the surface of 20 to ∼25 ml of complete liquid mCDB medium in a petri dish. After 36 h of incubation in darkness at 28°C, the mycelial mat was illuminated with white-light LED lamps. Control samples were harvested in complete darkness. Samples were frozen in liquid nitrogen and stored at −80°C until RNA isolation. Frozen mycelia were ground into powder and total RNA isolated using the E.Z.N.A. fungal RNA minikit (VWR). The isolated RNA was quantiﬁed and an aliquot treated with DNase I. RNA samples were diluted to a ﬁnal concentration of 50 ng/μl. Quantitative real-time PCR experiments were performed to determine relative mRNA abundance using the SensiFAST SYBR & No-ROX One-Step Kit from Bioline (Luckenwalde, Germany). Each reaction mixture of 25 μl contained 0.2 μl of reverse transcriptase (RT) enzyme, 0.2 μM primers, and 100 ng of total RNA. The cycle included 10 min at 50°C for the reverse transcription reaction, followed by 5 min at 95°C for its inactivation and 40 PCR cycles of 10 s at 95°C and 1 min at 60°C. After each PCR, we performed melting curve analyses to show the speciﬁc ampliﬁcation of single DNA segments and the absence of nonspeciﬁcally ampliﬁed DNA. The results for each gene were normalized to the corresponding results obtained with histone gene *H2B*. Then, the results obtained with each sample were normalized to the RNA sample obtained from wild-type mycelia in darkness or exposed to light for 30 min and are the average of the results from three to six biological replicates. For the oligonucleotides used in this study, see Table S1.

### Transcriptional profiling of osmotic and oxidative stress-associated genes.

To investigate osmotic and oxidative stress responses, transcripts of genes induced by osmotic and oxidative agents were analyzed in the cultures of mCDB supplemented or not with the agents. Prior to the addition of the stress agents, fresh spores were inoculated in a 50-ml flask and incubated at 28°C overnight. The culture was then supplemented with 0.7 M NaCl or 4 mM H_2_O_2_ and further shaken for 30 min. Total RNA isolation and real-time PCR with respective primers for the genes of interest were performed as described above.

### Immunofluorescence.

Fresh conidia were inoculated onto coverslips with 400 µl mCDB medium and cultivated for 3 h in the dark at room temperature. The samples were exposed to light or kept in the dark in chambers for 5 min and fixed immediately with 3.7% formaldehyde in phosphate-buffered saline (PBS) buffer for 30 min at room temperature. The coverslips with the adhered germlings were washed three times with PBS buffer and incubated with digestion solution (100 mg Driselase [Sigma-Aldrich], 20 µl of 5 U per ml Zymolase, and 800 mg glucan X diluted in 2.5 ml of 50 mM Na citrate [pH 5.8] and mixed with 2.5 ml egg white) for 1 h at room temperature. The coverslips were then washed three times with PBS buffer and blocked with 5% bovine serum albumin (BSA) in Tris-buffered saline with 0.1% Tween 20 (TBST) buffer for 30 min. Afterwards, germlings were incubated with antiphospho-p38 MAP kinase (Thr180/Tyr182) antibodies (no. 9211, 1:400 dilution; Cell Signaling Technology, Beverly, MA) in TBST buffer with 5% BSA overnight at 4°C and washed three times with TBST afterwards. Cy3-conjugated anti-rabbit IgG secondary antibody (Jackson Immunoresearch, West Grove, PA) was used at a 1:200 dilution in 5% BSA in TBST. After 1 h of incubation, the coverslips were washed three times with TBST and mounted on microscope slides for observation.

### Availability of data and materials.

All strains used in this study are available from the authors upon request.
