# Supplementary figures and images for: Red- and Blue-Light Sensing in the Plant Pathogen Alternaria alternata Depends on Phytochrome and the White-Collar Protein LreA
Source: mBio. 2019 Apr 9;10(2):e00371-19. doi: 10.1128/mBio.00371-19 (PMC6456751; doi:10.1128/mBio.00371-19)

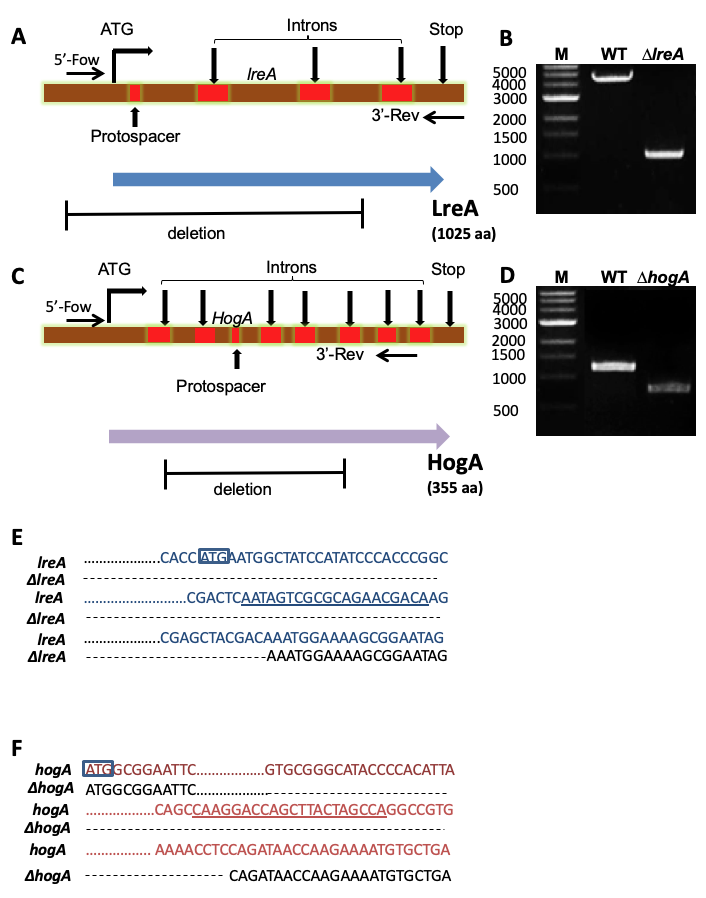

Supplement: FIG S1 [file mBio.00371-19-sf001.tif]

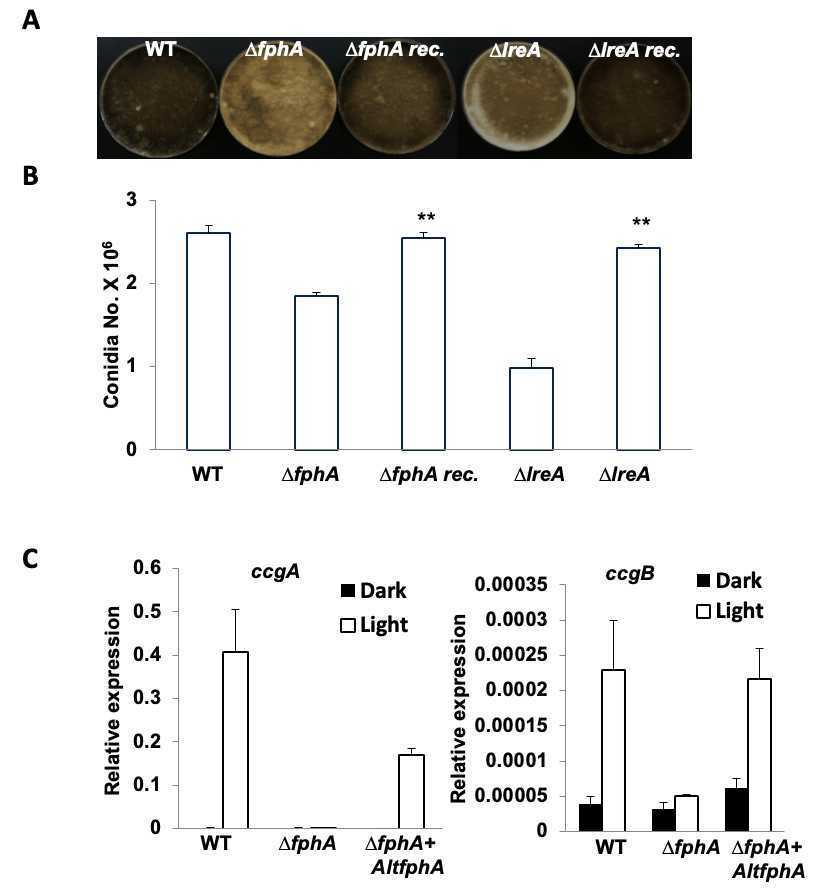

Supplement: FIG S2 [file mBio.00371-19-sf002.tif]

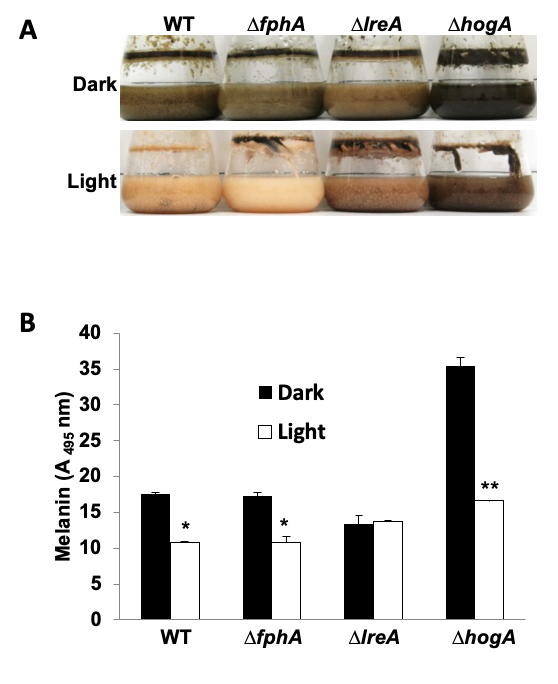

Supplement: FIG S3 [file mBio.00371-19-sf003.tif]

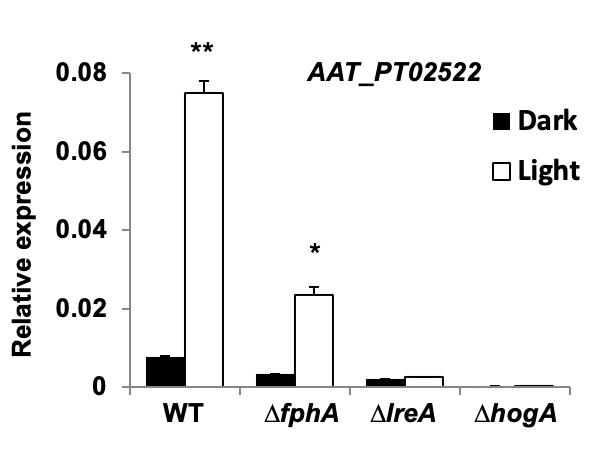

Supplement: FIG S4 [file mBio.00371-19-sf004.tif]

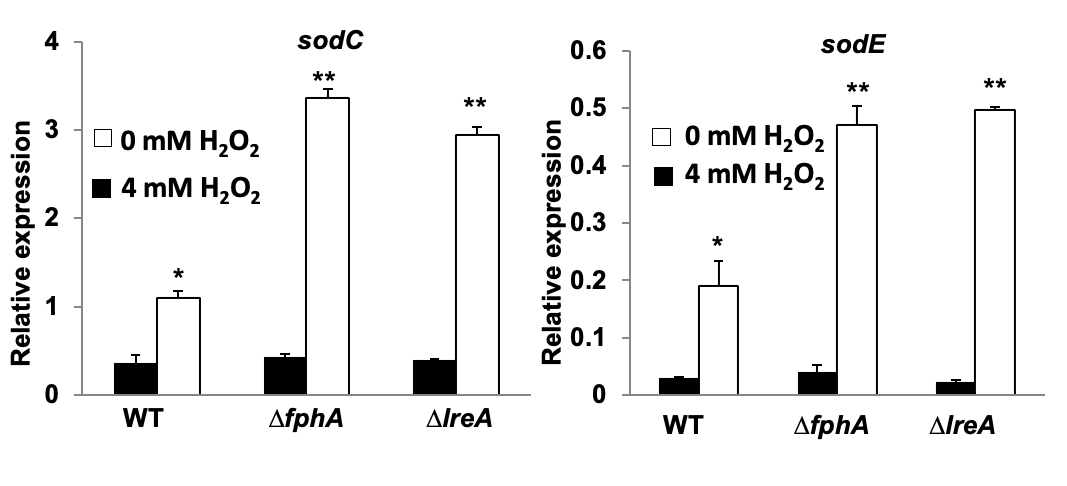

Supplement: FIG S5 [file mBio.00371-19-sf005.tif]

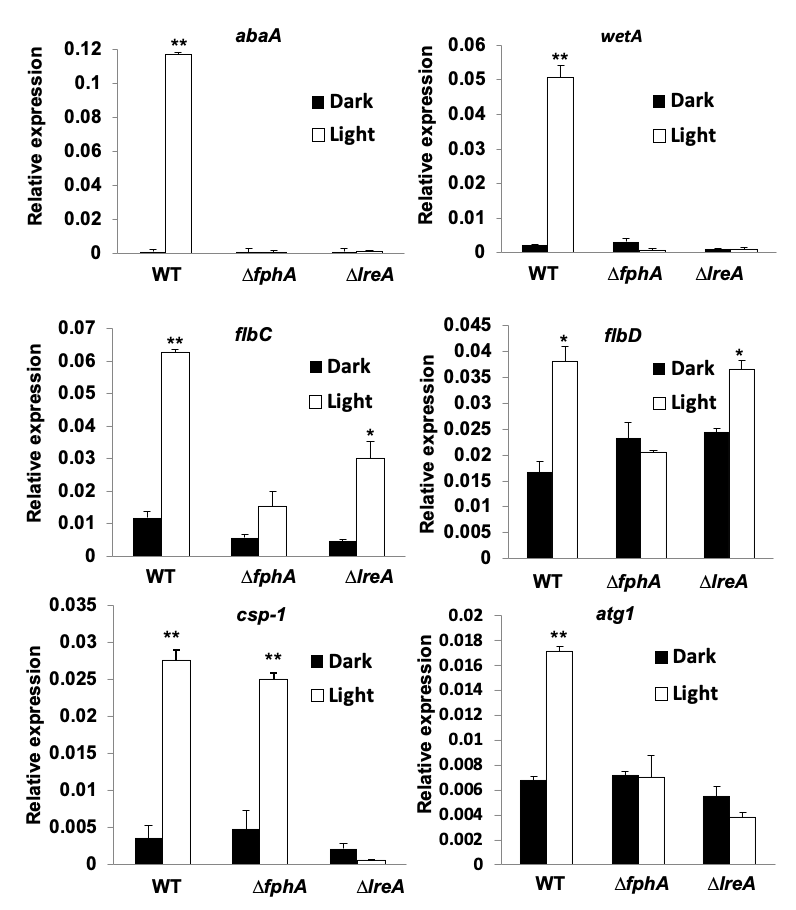

Supplement: FIG S6 [file mBio.00371-19-sf006.tif]
