# Supplementary material for: Red- and Blue-Light Sensing in the Plant Pathogen Alternaria alternata Depends on Phytochrome and the White-Collar Protein LreA
Source: mBio. 2019 Apr 9;10(2):e00371-19. doi: 10.1128/mBio.00371-19 (PMC6456751; doi:10.1128/mBio.00371-19)
Supplement: TABLE S1 [file mBio.00371-19-st001.docx]

**Suppl.** **Table 1: Oligonucleotides used in this study.** The red letters indicate the protospacer sequences.

| **Oligonucleotide** | **Sequence 5’ - 3’** |
| --- | --- |
| Crispy 2.0_fwd | GGTCATAGCTGTTTCCGCTGA |
| Crispy 2.0_rv | TGATTCTGCTGTCTCGGCTG |
| Proto_fphA_fwd | GTCCGTGAGGACGAAACGAGTAAGCTCGTCCAACTCTGGTCCTCCTCTACGTTTTAGAGCTAGAAATAGCAAGTTAAA |
| Hh_fphA_rv | GACGAGCTTACTCGTTTCGTCCTCACGGACTCATCACAACTCCGGTGATGTCTGCTCAAGCG |
| Proto_lreA_fwd | GTCCGTGAGGACGAAACGAGTAAGCTCGTCAATAGTCGCGCAGAACGACAGTTTTAGAGCTAGAAATAGCAAGTTAAA |
| Hh_lreA_rv | GACGAGCTTACTCGTTTCGTCCTCACGGACTCATCAAATAGTCGGTGATGTCTGCTCAAGCG |
| Proto_hogA_fwd | GTCCGTGAGGACGAAACGAGTAAGCTCGTCCAAGGACCAGCTTACTAGCCGTTTTAGAGCTAGAAATAGCAAGTTAAA |
| Hh_hogA _rv | GACGAGCTTACTCGTTTCGTCCTCACGGACTCATCACAAGGACGGTGATGTCTGCTCAAGCG |
| fphA_test_fw | CGTACTCTCGTCACGAGCAAG |
| fphA_test_rv | CTTGACGTCTAGTGCTTGGCT |
| lreA_test_fw | TCTGCTTGGCTGGGACATG |
| lreA_test_rv | GTGGCGGGATGAAGCCTT |
| hogA_test_fw | ATCGCATTTGGTGCCTGCC |
| hogA_test_rv | AGCATGATCTCAGGGGCTC |
| lreA_comp_fw | GACCGGTGTTTATCGTCTCAG |
| lreA_comp_rv | GAAGCGAAGCAAGGCAAGAC |
| fphA_comp_fw | TACTCTCGTCACGAGCAAGTG |
| fphA_comp_rv | TGACGTCTAGTGCTTGGCTTG |
| *A. nidulans* ccgA_RT_fw | CGACGCTTCCCTCACTTCTC |
| *A. nidulans* ccgA_RT_rv | CATCATGGGACTTCTCGTCCTT |
| *A. nidulans* ccgB_RT_fw | GGAGACTATCAAGGTAAGCATGTACC |
| *A. nidulans* ccgB_RT_rv | CTTGTCAAAGAGAGCGTCCTTG |
| *A. nidulans* h2B_RT_fw | CTGCCGAGAAGAAGCCTAGCAC |
| ccgA_RT_fw | GTCAACTCTGTCAAGAACGC |
| ccgA_RT_rv | TTGATCTTGTCACCAGCAGC |
| h2B_RT_fw | ACAAGAAGAAGCGCACCAAG |
| h2B_RT_rv | CGTTGACGAAAGAGTTGAGAA |
| fer_RT_fw | TGGACCCTATATTGCACGGAG |
| fer_RT_rv | GGTGTTCGGACCATTTCCTGA |
| bliC_RT_fw | GACCCCTACACGCAGAAGAA |
| bliC_RT_rv | GGTCAACAGGCAGAACTTGGT |
| AAT_PTO2522_RT_fw | GTCAACGGTGCTAAGGTGTAC |
| AAT_PTO2522_RT_rv | CCTGCAATGTTCTGACCATGC |
| hogA_RT_fw | CCTGAAATACGTCCACTCCG |
| hogA_RT_rv | GAGACCGAAGTCGCAAATCTG |
| atfA_RT_fw | CACCGTACACAACCCATTCTC |
| atfA_RT_rv | CTCCAGGTGTCTGCAAGTTTC |
| catA_RT_fw | GGCATTCTTACCGACACATCG |
| catA_RT_rv | TGTGTAGAACTTGACGGCGAAA |
| catD_RT_fw | CAACGTCTCCCTCGACAAG |
| catD_RT_rv | CAGTGAGAAGCATCAAGTCGG |
| sodA_RT_fw | ACACCACCATCTCATGGAACAT |
| sodA_RT_rv | CGTGTGTCTTGTTGTGGGGTT |
| sodB_RT_fw | GAGGCCAAGCAAAAGGAAGAC |
| sodB_RT_rv | GAAGAGGCTGTGGTTGATGTG |
| sodC_RT_fw | CATCAACCACTCGCTCTTCTG |
| sodC_RT_rv | GAACTTGTCCTCATCACCCC |
| sodE_RT_fw | GAGGCCAAGCAAAAGGAAGAC |
| sodE_RT_rv | GAAGAGGCTGTGGTTGATGTG |
| pksI_RT_fw | GGAAAACGTCACTTGGTGGA |
| pksI_RT_rv | TGTGCCTCTCGCAATTAGGA |
| abaA_RT_fw | CAAAAGTCACAACGTCACGGC |
| abaA_RT_rv | GTAGGTTCCTGTAGTGACGCA |
| wetA_ RT_fw | TACAATCAACTGAGGCACCGT |
| wetA_ RT_rv | AGCTGAAGGTTGGTGTTGAG |
| flbC_ RT_fw | CAATGGCAGTCGATAACGT |
| flbC_ RT_rv | GTGTGGTTGTTGTTGAGGGTA |
| flbD_ RT_fw | TCTGCATTGTTTTGGTGACCG |
| flbD_ RT_rv | GTCACAGCACGAGGATCAG |
| csp-1_RT_fw | GGTCGCAGAGTGTCATTGC |
| csp-1_RT_rv | CTGGAGGTCCTAGACTGGA |
| atg1_RT_fw | AGACCATGAGGAAAACGCCG |
| atg1_RT_rv | AATGCCTGGGACTCACGG |
|  |  |
